# An Inkling of Suspicion: Prenatal Exposure to PBDEs and Neurodevelopmental Impairment

**DOI:** 10.1289/ehp.118-a216a

**Published:** 2010-05

**Authors:** Kellyn S. Betts

**Affiliations:** **Kellyn S. Betts** has written about environmental contaminants, hazards, and technology for solving environmental problems for publications including *EHP* and *Environmental Science & Technology* for more than a dozen years

A longitudinal cohort study of more than 150 U.S. children conducted over 7 years associates prenatal exposure to higher concentrations of polybrominated diphenyl ether (PBDE) flame retardants with lower scores on tests of neurodevelopment **[*EHP*** **118:712–719; Herbstman et al.]**. This is the second recent epidemiologic study to link PBDEs with evidence of adverse effects on brain development, although differences in methodology between this and the other study [*EHP* 117:1953–1958; Roze et al.] make direct comparisons difficult.

PBDE flame retardants have been used for decades in a wide variety of goods, including automobile and airplane components, electronics, and home and office furnishings. The toxicologic evidence linking PBDEs to adverse health effects led the European Union to use the precautionary principle as the basis for banning all three PBDE formulations (penta, octa, and deca). In the United States, manufacturers voluntarily discontinued the penta and octa formulations in 2004 and have agreed to phase out deca by the end of 2012.

The mothers of the children in the current study were pregnant at the time of the World Trade Center (WTC) attacks in 2001 and gave birth at a hospital within 2 miles of the WTC site. The women were recruited for a study on the effects of exposure to compounds in dust from the decimated towers. PBDEs were measured in the cord blood of 210 infants, and 152 of these children later participated in at least one round of neurodevelopmental testing conducted at 1, 2, 3, 4, and 6 years of age.

The children with higher levels of exposures consistently had, on average, lower developmental scores at each time point compared with less-exposed children; the association was particularly evident at age 4 years. The researchers were not able to evaluate associations with developmental delay because few children had developmental scores low enough to meet the criterion for this outcome. However, in many cases average test scores in children with exposures in the highest 20% were 5–11 points lower than average scores for less-exposed children.

Although some evidence suggests the PBDE exposure seen in the children could be related to the WTC attack, the authors say “it is certain” that sources other than the WTC contributed to the PBDE levels in the infants’ cord blood. Because the levels observed in these children were similar to those reported in other U.S. populations, the new research suggests the observed effects could be widespread.

According to the authors, these findings are consistent with reports of hyperactivity and learning and memory deficits in experiments with mice exposed neonatally to PBDEs. Other work, most recently a laboratory study using human cells [*EHP* 118:572–578; Schreiber et al.], suggests PBDEs may interfere with thyroid hormones critical for normal brain development. The authors point out that additional studies exploring associations between PBDE exposure and developmental effects are under way. In the meantime, they say identifying opportunities to reduce people’s exposure to these compounds should be a priority.

